# Biomass Smoke Exposure Enhances Rhinovirus-Induced Inflammation in Primary Lung Fibroblasts

**DOI:** 10.3390/ijms17091403

**Published:** 2016-08-25

**Authors:** Sarah J. Capistrano, Razia Zakarya, Hui Chen, Brian G. Oliver

**Affiliations:** 1 Molecular Biosciences, School of Life Sciences, University of Technology Sydney, Sydney, NSW 2007, Australia; Sarah.Capistrano@student.uts.edu.au (S.J.C.); Razia.zakarya@student.uts.edu.au (R.Z.); Hui.Chen-1@uts.edu.au (H.C.); 2 Respiratory Cellular and Molecular Biology, Woolcock Institute of Medical Research, Sydney, NSW 2037, Australia

**Keywords:** Chronic obstructive pulmonary disease, Biomass smoke, Rhinovirus

## Abstract

Biomass smoke is one of the major air pollutants and contributors of household air pollution worldwide. More than 3 billion people use biomass fuels for cooking and heating, while other sources of exposure are from the occurrence of bushfires and occupational conditions. Persistent biomass smoke exposure has been associated with acute lower respiratory infection (ALRI) as a major environmental risk factor. Children under the age of five years are the most susceptible in developing severe ALRI, which accounts for 940,000 deaths globally. Around 90% of cases are attributed to viral infections, such as influenza, adenovirus, and rhinovirus. Although several epidemiological studies have generated substantial evidence of the association of biomass smoke and respiratory infections, the underlying mechanism is still unknown. Using an in vitro model, primary human lung fibroblasts were stimulated with biomass smoke extract (BME), specifically investigating hardwood and softwood types, and human rhinovirus-16 for 24 h. Production of pro-inflammatory mediators, such as IL-6 and IL-8, were measured via ELISA. Firstly, we found that hardwood and softwood smoke extract (1%) up-regulate IL-6 and IL-8 release (*p* ≤ 0.05). In addition, human rhinovirus-16 further increased biomass smoke-induced IL-8 in fibroblasts, in comparison to the two stimulatory agents alone. We also investigated the effect of biomass smoke on viral susceptibility by measuring viral load, and found no significant changes between BME exposed and non-exposed infected fibroblasts. Activated signaling pathways for IL-6 and IL-8 production by BME stimulation were examined using signaling pathway inhibitors. p38 MAPK inhibitor SB239063 significantly attenuated IL-6 and IL-8 release the most (*p* ≤ 0.05). This study demonstrated that biomass smoke can modulate rhinovirus-induced inflammation during infection, which can alter the severity of the disease. The mechanism by which biomass smoke exposure increases inflammation in the lungs can be targeted and inhibited via p38 MAP kinase pathway.

## 1. Introduction

Biomass smoke is the result of the combustion of different types of biomass fuels, such as wood, animal dung, crop residues, and coal generated by more than 3 billion people for cooking and heating. As such, biomass smoke is one of the major air pollutants and contributors of household air pollution worldwide. In developing countries where poverty is prevalent, burning biomass fuels is a cheaper alternative compared to liquefied petroleum gas (LPG) or electricity. Biomass fuels are also more accessible, especially for people living rurally [1]. As much as 97% of the population, living in rural areas in the least developed countries, rely solely on biomass fuels for household energy purposes [2].

Several studies have shown that women and children have the highest biomass smoke exposure due to cultural practices, such as indoor cooking in housing with very poor air ventilation [3]. The absence of chimneys or pipes prevents the smoke from venting outside and, therefore, particles become trapped and fuse to the surroundings [4,5]. During the burning of these fuels, people indoors can be exposed with up to 30,000 µg/m^3^ of particulate matter (PM) sized 10 µm or smaller (PM_10_), while an average concentration throughout the day is approximately 300–5000 µg/m^3^. Since most women and children stay indoors, they are exposed to these high concentrations of particulate matter and other toxic air pollutants for about 3–7 h a day [6]. The WHO guideline for PM10 concentration is only 50 µg/m^3^ for a 24-h period. The Global Burden of Disease 2010 study found that household air pollution is the second highest risk factor of ill health for women and girls globally [7].

Children exposed to biomass smoke have an increased risk (1.8-fold) of developing acute lower respiratory disease (ALRI) [8,9]. Younger children, especially aged five years or younger, are more susceptible and have a higher mortality rate. ALRI accounts for 940,000 deaths of children under five per year [10]. Biomass smoke-associated ALRI causes 455,000 deaths and a loss of 39.1 million disability-adjusted life years annually [5,11]. However, diagnosis of ALRI is often based on parent-reported symptoms which can compromise etiological specificity [4]. Generally, ALRI is characterized by acute bronchitis, bronchiolitis, and pneumonia, caused by respiratory bacterial or viral infections. Around 90% of cases are attributed to viral infections, such as influenza, adenovirus, and rhinovirus. Although epidemiological studies have shown that biomass smoke is a considerable risk factor for the development of ALRI, the exact mechanism of the resulting increase in susceptibility is still unknown. Therefore, this study aims to investigate the effect of biomass smoke exposure on primary human lung cells in vitro, specifically examining possible enhancement of respiratory infection such as rhinovirus, by measuring production of inflammatory mediators involved in immune responses against infection.

## 2. Results

### 2.1. Hardwood and Softwood Smoke Extract Are Cytotoxic at Higher Concentrations

**Figure 1:**
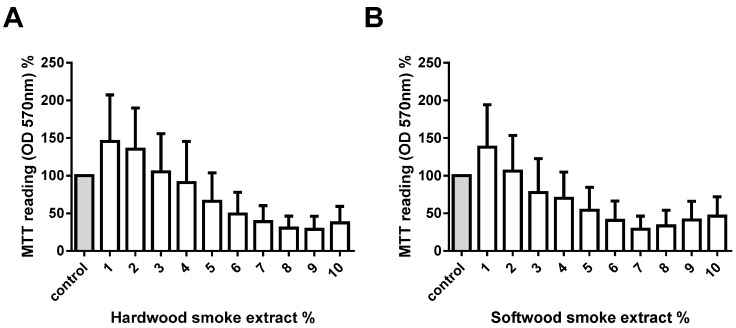
Measurement of cell viability with different hardwood and softwood smoke extract concentrations. Human primary lung fibroblasts (*n* = 5) were stimulated with hardwood (**A**) and softwood (**B**) smoke extract (1%–10%) in 0.1% FBS/DMEM. Cell viability was measured using MTT assay at 24 h after stimulation. Data expressed as the percent of unstimulated fibroblasts and bars represent mean ± SEM. Statistical analysis was executed using one-way ANOVA with Tukey’s post-test. No significant differences were found.

**Figure 2:**
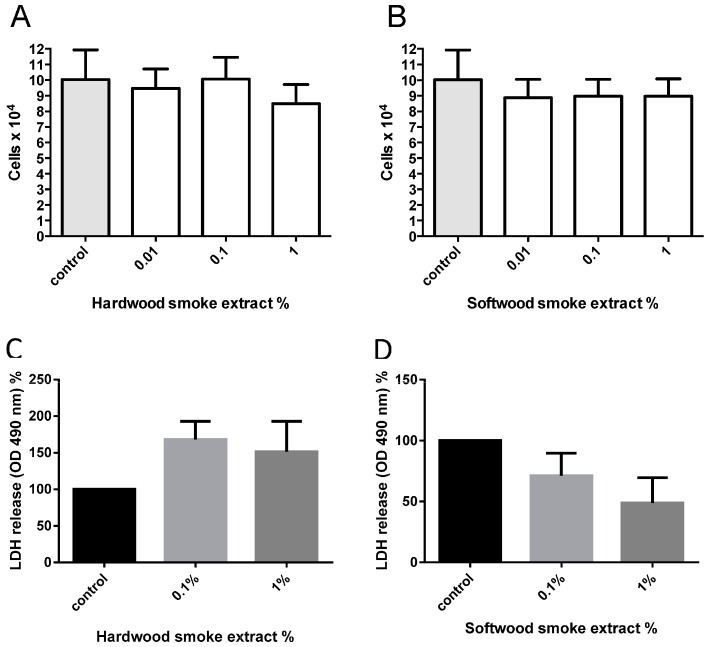
Measurement of cell viability from hardwood (**A**,**C**) and softwood (**B**,**D**) smoke extract stimulation at lower concentrations. Cell viability was measured via Manual cell count with trypan blue (0.02% *w*/*v*) and LDH assay from hardwood and softwood smoke extract stimulation at 0.01%, 0.1%, and 1% diluted in 0.1% FBS/DMEM in primary human lung fibroblasts (*n* = 6). Manual cell count and LDH assay was executed after 24 h post-stimulation. Data is expressed in cells/mL (**A**,**B**), percent of LDH release from control (**C**,**D**), and bars represent mean ± SEM. Comparison between cell counts from control and different concentrations of hardwood and softwood smoke extract stimulation made by one-way ANOVA with Tukey’s post-test. No significant differences were found.

To assess the potential toxicological effect of biomass smoke, cells were exposed to 1%–10% of hardwood and softwood smoke for 24 h stimulation. Cell viability was measured via MTT assay, and confirmed by trypan blue exclusion assays. We found a trend of decreasing number of viable cells with increasing concentrations of hardwood and softwood smoke extract stimulation (Figure 1). We then investigated lower concentrations (0.01%, 0.1%, and 1%) of hardwood and softwood smoke extract and found no toxic effects of biomass smoke extract by trypan blue exclusion assays (Figure 2A,B) and LDH assays (Figure 2C,D). Overall we observed no differences in the toxicity of hardwood and softwood smoke extract.

### 2.2. Hardwood and Softwood Smoke Extract Upregulates IL-6 and IL-8 Production at Low Concentrations

**Figure 3:**
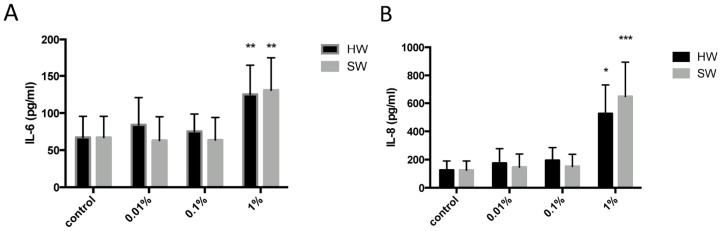
IL-6 (**A**) and IL-8 (**B**) induction from Hardwood and Softwood smoke exposure at lower concentrations. Human primary lung fibroblasts (*n* = 6) were stimulated with hardwood and softwood smoke extract (0.01%, 0.1% and 1%) in 0.1% FBS/DMEM for 24 h. Cell free supernatants were collected and IL-6 (**A**) and IL-8 (**B**) release was measured via ELISA. Data were expressed in pg/mL and bars represent mean ± SEM. Comparisons between IL-6/IL-8 release from control and different concentrations of hardwood and softwood smoke extract made by one-way ANOVA with Tukey’s post-test. Significance is represented as * *p* < 0.05, ** *p* < 0.01 vs. control, *** *p* < 0.001 vs. control.

Cell free supernatants were collected from fibroblasts stimulated with hardwood and softwood smoke extract (0.01%, 0.1%, and 1%) and IL-6 and IL-8 release was assessed via ELISA. We found a significant increase of both IL-6 and IL-8 release from 1% hardwood and softwood smoke extract stimulation (Figure 3).

### 2.3. Biomass Smoke Exposure Enhances RV-16 Induced IL-8 Production

**Figure 4:**
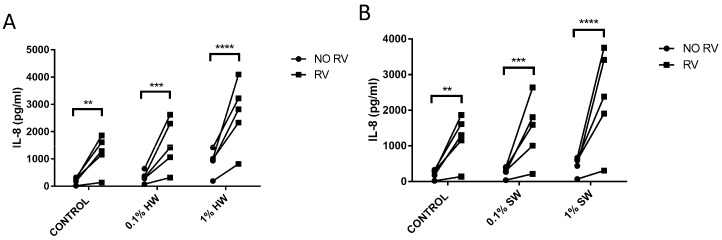
Measurement of IL-8 production from hardwood (**A**) and softwood (**B**) smoke exposure and RV-16 infection. Primary human lung fibroblasts (*n* = 5) were stimulated with hardwood and softwood smoke extract at 0.1% and 1% concentration alone, RV-16 infection alone (MOI = 1), or both, and incubated for 24 h. Unstimulated fibroblasts were also measured for IL-8 constitutive release. Supernatants were collected for IL-8 concentration analysis via ELISA. Data expressed as pg/mL. Statistical analysis was executed using two-way ANOVA with Sidak’s post-test. Significance is represented as ** *p* < 0.01, *** *p* < 0.001, **** *p* < 0.0001.

**Figure 5:**
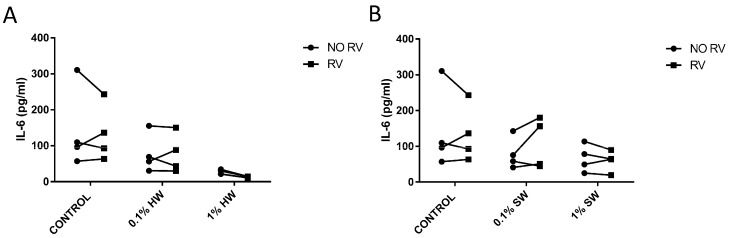
Measurement of IL-6 production from hardwood (**A**) and softwood (**B**) smoke exposure and RV-16 infection. Primary human lung fibroblasts (*n* = 4) were stimulated with hardwood and softwood smoke extract at 0.1% and 1% concentration alone, RV-16 infection alone (MOI = 1), or both and incubated for 24 h. Unstimulated fibroblasts were also measured for IL-6 constitutive release. Supernatants were collected for IL-6 concentration via ELISA. Data expressed as pg/mL. Statistical analysis was executed using two-way ANOVA with Sidak’s post-test.

Since epidemiological evidence suggests an interaction between biomass smoke and viral infection, we modelled this interaction in vitro. Fibroblasts were stimulated with biomass smoke extract initially (0.1% or 1%) and the infected with RV-16. As expected, hardwood and softwood smoke extract, and RV-16 alone, induced IL-6 and IL-8 release. Interestingly, RV increased IL-8 (Figure 4), but not IL-6 production (Figure 5) in both hardwood and softwood smoke exposed fibroblasts. In cells first infected with RV and then stimulated with biomass smoke extract, cytokine induction was not greater in comparison to RV alone.

### 2.4. Biomass Smoke Exposure Did Not Affect RV-16 Load

We further investigated the role of biomass smoke exposure on modulating RV-16 infection by measuring viral load of infected fibroblasts with or without biomass smoke exposure. There were no significant differences between the viral load of biomass smoke exposed and non-exposed RV-16 infected fibroblasts (Figure S1). We also measured viral load from RV-16 infected fibroblasts that were exposed to biomass smoke extract after infection and found no significant difference between the groups (data not shown).

### 2.5. Hardwood and Softwood Smoke Induced IL-6 and IL-8 Are Attenuated by Different Signalling Pathway Inhibitors

**Figure 6:**
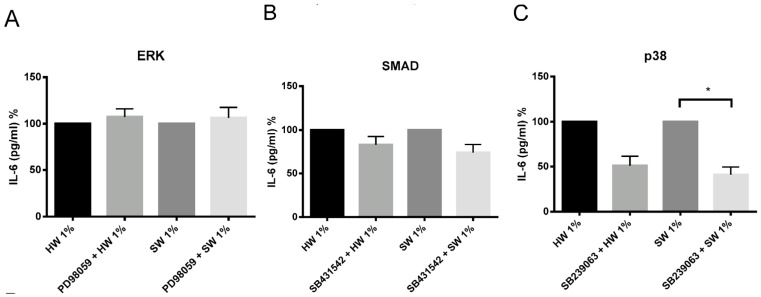
The effects of ERK (**A**), SMAD (**B**), and p38 MAP kinase (**C**) inhibitors on IL-6 production by hardwood and softwood smoke extract. Primary human lung fibroblasts (*n* = 4) were pretreated for an hour with signaling inhibitors, PD98059 (10 µM), SB431542 (10 µM), and SB239063 (3 µM) in DMSO (vehicle control) for ERK, SMAD, and p38 MAP kinase pathway, respectively, then stimulated with 1% hardwood and softwood smoke extract for 24 h. Supernatant was collected and IL-6 concentration analysis was executed via ELISA. Data are expressed as the percent of inhibition from control and bars represent mean ± SEM. Statistical analysis was executed by using repeated measures, one-way ANOVA with Tukey’s post-test. Significance is represented as * *p* < 0.05.

**Figure 7:**
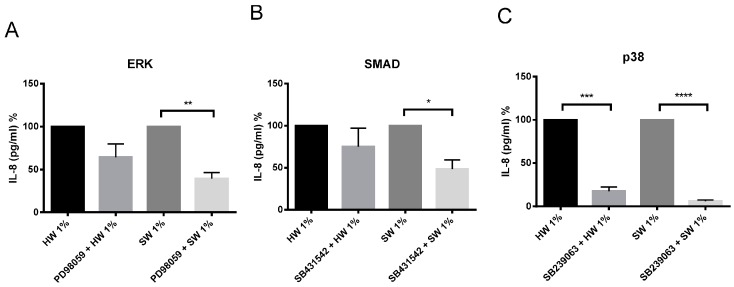
The effects of ERK (**A**), SMAD (**B**), and p38 MAP kinase (**C**) inhibitors on IL-8 production by hardwood and softwood smoke extract. Primary human lung fibroblasts (*n* = 4) were pretreated for an hour with signaling inhibitors, PD98059 (10 µM), SB431542 (10 µM), and SB239063 (3 µM) in DMSO (vehicle control) for ERK, SMAD, and p38 MAP kinase pathway respectively, then stimulated with 1% hardwood and softwood smoke extract for 24 h. Supernatant was collected and IL-8 concentration analysis was executed via ELISA. Data expressed as the percent of inhibition from control and bars represent mean ± SEM. Statistical analysis executed by using repeated measures, one-way ANOVA with Tukey’s post-test. Significance is represented as * *p* < 0.05, ** *p* < 0.01, *** *p* < 0.001, **** *p* < 0.0001

There are different potential signaling pathways being activated by biomass smoke to induce IL-6 and IL-8 production. PI3 kinase, ERK, SMAD, p38 MAP kinase, NFκB, JNK and COX signaling pathways have all been previously shown to play a role in up-regulating inflammation from oxidative stimuli such as cigarette smoke. To examine this, chemical inhibitors were used, which are specific to the signaling pathways mentioned above. PI3 kinase, NFκB, JNK, and COX inhibitors were unable to attenuate IL-6 and IL-8 production from both hardwood and softwood smoke extract stimulation (data not shown). Softwood smoke-induced IL-6 and IL-8 was significantly attenuated by p38 MAP kinase inhibitor (Figure 6 and Figure 7). In addition, softwood smoke-induced IL-8 was also inhibited by ERK and SMAD inhibitors (Figure 7). Surprisingly, hardwood smoke-induced IL-6 was not inhibited by any of the signaling inhibitors used (Figure 6), however, hardwood smoke-induced IL-8 solely involved p38 MAP kinase pathway (Figure 7).

## 3. Discussion

As expected, this study found that hardwood and softwood smoke extract up-regulates IL-6 and IL-8 release from primary human lung fibroblasts, suggesting that biomass smoke exposure promotes airway inflammation. Interestingly, in the presence of biomass smoke, rhinovirus (RV) was able to further increase IL-8 production, but had no effect on IL-6 production. Biomass smoke exposure did not affect RV replication. To understand the underlying mechanisms involved in biomass smoke-induced IL-6 and IL-8 production, activated signaling pathways were also investigated by using signaling pathway inhibitors. Despite relatively similar toxicity and cytokine induction, the signaling pathways activated by softwood and hardwood biomass smoke were very different. IL-6 production is mainly driven by p38 MAPK. However, softwood smoke extract induced IL-8 via SMAD, ERK, and p38 MAPK, whilst hardwood extract induced IL-8 via p38 MAPK only.

The assessment of cellular toxicology to hardwood and softwood smoke was made using a number of biochemical and biological assays. The aim of these experiments was to choose a concentration of biomass smoke that was non-toxic for further immunogenic analysis. For screening purposes, we chose to use a MTT assay with biomass smoke at 1%–10%. The MTT assay measures mitochondrial activity and not cellular viability, per se. The assay overall showed no statistical change, but a trend towards increased MTT with low concentrations of biomass extract and reduced MTT with higher concentrations was observed. The increased MTT readings could represent either proliferation or increased mitochondrial biogenesis, and the lower readings reduced number of cells or mitochondria. To investigate this further, we chose to count the actual number of viable cells over the concentration range 0.01%–1% biomass smoke extract. No increase or decrease in cell number was found, leading us to assume that the trend towards increased MMT readings at 1% biomass smoke extract is the result of greater mitochondrial activity and/or number. To thoroughly investigate if cell viability was compromised by 1% biomass extract we carried out LHD assays. No significant release of LDH occurred with biomass extract stimulation, although the data were variable and a trend towards increased LDH with hardwood smoke and decreased LDH with softwood smoke extract was observed. These data do have limitations. The gold standard assay of cellular viability is direct microscopic assessment with an exclusion dye, such as trypan blue. The MTT assay and the LDH assay could both be effected by the presence of oxidants, particulate matter, and other chemicals in hardwood and softwood smoke extract. We are confident that biomass smoke at 1% is nontoxic, but are not certain what the potential changes in MTT or LDH measurements represent.

This study is the first to investigate the interaction of biomass smoke exposure and rhinovirus infection in vitro. Epithelial cells and fibroblasts are often the first non-immune cells within the respiratory tract to encounter both viral pathogens and toxic components of air and are essential for innate and subsequent adaptive immune response. Upon RV infection, the cells produce various cytokines and chemokines, such as IL-6 and IL-8 [12] that are capable of activating and recruiting a variety of other cells such as lymphocytes, eosinophils, and neutrophils [13]. We found that prior biomass smoke exposure enhances rhinovirus-induced IL-8 production. As IL-8 production in vivo is known to be positively correlated to respiratory symptoms in rhinovirus infected children [14]. We believe that this enhanced secretion of IL-8 may, in part, explain the enhanced severity of virus infections in biomass smoke exposed people.

It was interesting that rhinovirus replication was not altered in biomass smoke exposed fibroblasts. This suggests that the oxidative environment of the biomass smoke extract does not affect the virus capsid enough to alter infection. As IL-8 can be induced by RV-16, independent of viral replication, intracellular adhesion molecule (ICAM) [15], we cannot definitively state that RV-induced IL-8 occurred as a consequence of replication. However, it is reasonable to assume that IL-8 production was in-part induced by RV replication as replication occurred, even in the presence of biomass smoke extract.

Biomass smoke, specifically wood smoke, has been well characterized of its chemical and physical composition. There are over 200 different compounds, including toxic chemicals, such as polycyclic aromatic hydrocarbons (PAH), nitrogen oxides (NO_x_), and particulate matter of varying sizes (e.g., PM_2.5_, _10_). These compounds are well studied and are toxicologically indistinguishable despite different wood sources. Since we observed similar responses in hardwood and softwood smoke extract stimulated fibroblasts, we did not attempt to identify specific compounds in biomass smoke extract responsible for the changes evident in our study. It is most likely that these different compounds simultaneously contribute to the aftermath of physiological changes involved; therefore, it is more beneficial to study the entire biomass smoke as a physiological stimulus, similar to other studies involving cigarette smoke. The majority of the toxic components identified in biomass smoke are also present in cigarette smoke and, hence, both stimuli can be comparable in causing similar diseases. Several studies have shown increased susceptibility to ALRI in children from second-hand tobacco smoke [16,17,18]. Proud et al. have shown an additive increase of IL-8 production from cigarette smoke and rhinovirus stimulation in primary human bronchial epithelial cells, parallel to our findings with biomass smoke [19]. However, current publications have conflicting data on the effect of cigarette smoke on viral replication. Several studies have shown increased rhinovirus replication after cigarette smoke exposure compared to control [20,21,22]. However, other studies infecting the airway epithelial cell line BEAS-2B with RV-16 [19,23] reported that addition of CSE during and after infection had no effect on viral titer which is consistent with our results with biomass smoke exposure. Since this is the first study to investigate the interaction between biomass smoke exposure and rhinovirus infection, we can only compare our results to studies investigating cigarette smoke exposure. More research on biomass smoke exposure in cell culture models is crucial to understand mechanisms in causing and increasing risk in disease.

We have used an in vitro model to attempt to replicate the effects of exposure to biomass smoke in vivo. In women exposed to biomass smoke the equivalent exposure to cigarette is calculated as 20 cigarettes per day. In our model system we use the same mass of biomass as tobacco leaf found in a cigarette. However, cigarettes are directly inhaled whilst this is not the intention with biomass smoke. If we assume that 5 kg of wood would be used as a biomass heating source, our model of 1% biomass smoke extraction (from <1 g of wood), would equate to 1/500,000 of the total particles emitted from the 5 kg of biomass wood. We think that this is a reasonable approximation for what might occur in vivo, but acknowledge that room size, ventilation and breathing rate would all effect exposure. Interestingly, peak indoor particulate matter (PM10) whilst cooking often exceeded 2 mg/m^3^ [24].

Our model of biomass smoke extract does have advantages over direct smoke exposure. For example, it is easier to standardize exposure in comparison to direct smoke exposure, and when modelling effects on cells distal to the epithelium may be a closer approximation to what these cells are exposed to. However, it does have limitations. Volatile organic compounds (VOCs) are produced during biomass combustion. As we collect the soluble component of biomass smoke, it is possible that some VOCs will not be collected due to limited solubility and/or if collected they may not be present at the time of stimulation due to their short half-life. It would be interesting in future studies to look at the relative and separate contribution of particulate matter and VOCs in in vitro and in vivo models.

This study also investigated the possible differences between hardwood and softwood as two types of biomass source. Previous studies have characterized smoke compositions between different woods, and have found that the greatest variation occurs between hard wood and soft wood, rather than between different types of either. Hardwood and softwood have varying levels of resin acid and substituted phenols. More specifically, resin acid is more prominent in softwood types than hardwood [25]; and polycyclic aromatic hydrocarbon profiles between hardwood and softwood [26] Our study shows that 1% hardwood and softwood smoke extract both induce IL-6 and IL-8 release in primary human lung fibroblasts, and is cytotoxic at higher concentrations.

We also investigated the underlying mechanism involved in hardwood and softwood-induced IL-6 and IL-8 production by identifying activated signaling pathways using specific signaling inhibitors. p38 MAPK inhibitor SB239063 significantly attenuated IL-6 and IL-8 release the most. Interestingly, hardwood smoke extract might only be activating the p38 MAP kinase pathway to induce IL-8 production, while softwood smoke extract activates ERK, SMAD, and p38 MAP kinase pathways simultaneously to induce IL-8 production. These data have limitations. The use of specific pathway inhibitors is a good indication of the involvement of a specific pathway in a given response, but measurement of protein phosphorylation, translocation and binding to gene promoter regions is needed to confirm the direct involvement of the pathway. Despite similar cytotoxic and inflammatory effects of hardwood and softwood, different mechanisms might be involved in promoting inflammation. This suggests that the effect of biomass smoke can be source-specific and potentially require different therapeutic targets to inhibit inflammation.

## 4. Materials and Methods

### 4.1. Ethics Statement

Ethical approval for all experiments with lung tissue from donors undergoing resections or transplantations provided by the Human Ethics Committees of the University of Sydney and the Sydney South West Area Health Service with written consent forms.

### 4.2. Isolation and Culture of Primary Human Lung Fibroblasts

Primary human lung fibroblasts were isolated from lung tissue resection for thoracic malignancies or transplantation for interstitial lung disease or emphysema (mean age 63) as previously stated [27]. There were no available data on donor’s exposure to environmental pollution or biomass smoke prior to sample collection, although this was unlikely to be significant in Australian donors.

### 4.3. Cell Culture

Primary human lung fibroblasts were seeded at 4.2 × 10^4^ cells/mL in 5% FBS DMEM, at 37 °C with 5% CO_2_ for 72 h. Cells were synchronized into G0 phase in 0.1% FBS DMEM for 24 h before stimulation.

### 4.4. Biomass Smoke Extract Preparation and Stimulation

Two types of biomass were used in these experiments representing hardwood and softwood. The hardwood source was Merbau wood (*Intsia bijuga*), while the softwood source was standard pine (*Pinus radiata*). Both are commonly found in the Indo-pacific region and Asia where biomass smoke exposure is most prevalent. Both wood sources were untreated.

Biomass smoke extract (BME) was prepared fresh by using a custom built smoking apparatus, which allows the soluble components of biomass smoke to be collected in media. 730 mg of shredded biomass (hardwood/softwood) was combusted and the resulting biomass smoke was bubbled through 25 mL of DMEM, this was defined as 100% BME solution. This solution was then diluted in 0.1% FBS/DMEM to the desired concentration for experiments and used within 30 min after preparation.

### 4.5. ELISA Detection of IL-6 and IL-8

The concentration of IL-6 and IL-8 in cell free samples was measured using commercial ELISA kits specific for human IL-6 and IL-8 (BD Biosciences, North Ryde, Australia) and ELISA plates (NUNC Maxisorp, Naperville, IL, USA). All antibodies were used at concentrations recommended by manufacturer’s instructions and the following protocol was used.

### 4.6. Cell Viability Assay

Cell viability was estimated using 3-(4,5-Dimethylthiazol-2-yl)-2,5-Diphenyl-tetrazolium Bromide (MTT) assays and lactate dehydrogenase assays according to the manufacturer’s instructions (Sigma-Aldrich, St. Louis, MO, USA). In addition, manual cell counting with Trypan blue exclusion (0.02% *w*/*v*) was performed to confirm cell viability results.

### 4.7. Viral Propagation, Titration, and Stimulation

Major group human rhinovirus serotype-16 (RV-16) was obtained from Johnston at Imperial Ccollege, UK and propagated and titrated in Ohio HeLa cells [28]. Cells were infected with RV-16 (MOI of 1) after the medium was replaced with fresh 0.1% FBS/DMEM. Viral load was also measured from cell free supernatant samples collected from primary human lung fibroblasts that were infected with RV-16 alone, and with hardwood or softwood smoke extract stimulation using titration assay [28].

### 4.8. Biomass Smoke Extract and Human Rhinovirus-16 Stimulation

Primary human lung fibroblasts were stimulated with either 0.1% or 1% hardwood or softwood smoke extract alone for 24 h, or with RV-16 infection. Cells were either first stimulated with biomass smoke or infected for 4 h, and then the second stimulus (biomass or RV as appropriate) for another 20 h.

### 4.9. Signalling Pathway Inhibition Using Specific Inhibitors

To investigate the possible signaling pathways activated by biomass and resulting in cytokine production in primary human lung fibroblasts, inhibitors of the PI3 kinase, ERK, JNK, SMAD, p38 MAP kinase, NFκB, and COX pathways were used. The signaling pathway inhibitors were used at the following concentrations which have been shown to be specific and effective in human airway cells [29,30,31,32,33]—L294002 (3 μM), PD98059 (10 μM), SP600125 (10 μM) (Chalbiochem, San Diego, CA, USA), SB431542 (10 μM), SB239063 (3 μM) (Tocris, Ellisville, MO, USA), BAY117085 (10^−3^ μM), and acetylsalicylic acid (0.1 μM) (Sigma-Aldrich).

Signaling inhibitors were added to primary human lung fibroblasts for one hour prior to biomass smoke stimulation. A vehicle control (0.06% DMSO) was also used. Supernatant was collected after 24 h.

### 4.10. Statistical Analysis

Statistical analysis was performed using Graph Pad Prism (version 6, La Jolla, CA, USA). Data were initially checked for normality. To assess statistical significance, One-way repeated measures ANOVA with Tukey’s post-test were used for unpaired data. Two-way repeated measures ANOVA with Sidak’s post-test were used for paired data. All data on bar graphs were presented as mean ± standard error of the mean (SEM). Significance was shown with * for *p* value ≤ 0.05, ** for *p* ≤ 0.01, *** for *p* ≤ 0.001, and **** for *p* ≤ 0.0001.

## Supplementary Materials

Supplementary materials can be found at www.mdpi.com/1422-0067/17/9/1403/s1.
