# Supplementary material for: Biomass Smoke Exposure Enhances Rhinovirus-Induced Inflammation in Primary Lung Fibroblasts
Source: Int J Mol Sci. 2016 Aug 25;17(9):1403. doi: 10.3390/ijms17091403 (PMC5037683; doi:10.3390/ijms17091403)
Supplement: Supplementary file 1 [file ijms-17-01403-s001.pdf]

Supplementary Materials: Biomass Smoke Exposure Enhances Rhinovirus-Induced Inflammation in Primary Lung Fibroblasts

Sarah J. Capistrano, Razia Zakarya, Hui Chen and Brian G. Oliver

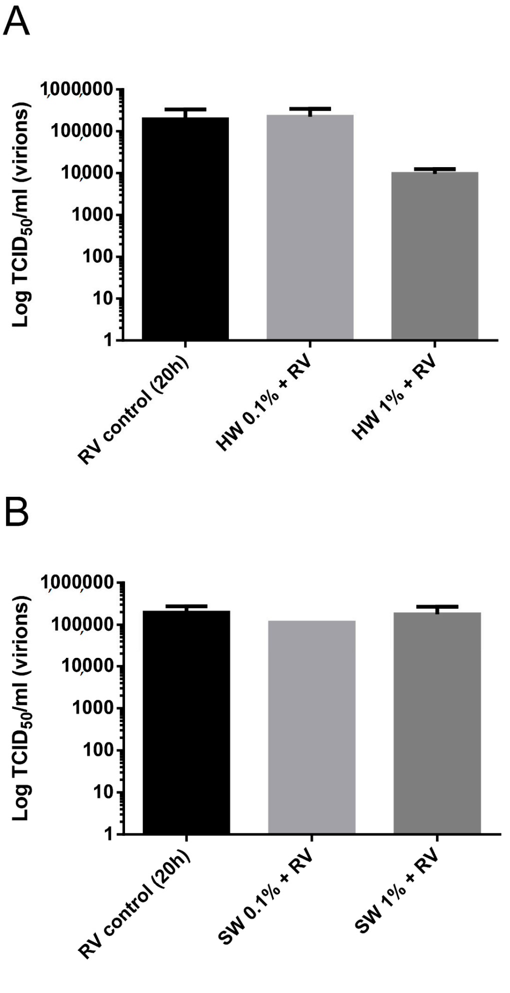

Figure S1. Measurement of viral load from RV-16 infected primary human lung fibroblasts stimulated with Hardwood (**A**) and Softwood (**B**) smoke extract. Fibroblasts were infected with RV-16 and either exposed to hardwood smoke extract (0.1%, 1%) or no exposure for 24 h. Cell free supernatants were collected and viral load was measured via viral titration assay. Data presented as mean ± SEM. Statistical analysis executed using One-way ANOVA with Tukey's post-test. No-significant differences were found, *n* = 3.
